# MiRNA Targeted NP Genome of Live Attenuated Influenza Vaccines Provide Cross-Protection against a Lethal Influenza Virus Infection

**DOI:** 10.3390/vaccines8010065

**Published:** 2020-02-03

**Authors:** Feixia Gao, Tianhan Yang, Xueying Liu, Feifei Xiong, Jian Luo, Yinglei Yi, Jiangfeng Fan, Ze Chen, Wen-Song Tan

**Affiliations:** 1 Department of Virus and Vaccine, Shanghai Institute of Biological Products, Shanghai 200052, China; drgaofeixia@foxmail.com (F.G.);; 2 State Key Laboratory of Bioreactor Engineering, School of Bioengineering, East China University of Science and Technology, Shanghai 200237, China

**Keywords:** miRNA-192-5p, influenza virus, NP, live attenuated influenza vaccine, cross-protection

## Abstract

The miRNA-based strategy has been used to develop live attenuated influenza vaccines. In this study, the nucleoprotein (NP) genome segment of the influenza virus was inserted by different perfect miRNA-192-5p target sites, and the virus was rescued by standard reverse genetics method, so as to verify the virulence and protective efficacy of live attenuated vaccine in cells and mice. The results showed there was no significant attenuation in 192t virus with one perfect miRNA-192-5p target site, and 192t-3 virus with three perfect miRNA target sites. However, 192t-6 virus with 6 perfect miRNA target sites and 192t-9 virus with 9 perfect miRNA target sites were both significantly attenuated after infection, and their virulence were similar to that of temperature-sensitive (TS) influenza A virus (IAV) which is a temperature-sensitive live attenuated influenza vaccine. Mice were immunized with different doses of 192t-6, 192t-9, and TS IAV. Four weeks after immunization, the IgG in serum and IgA in lung homogenate were increased in the 192t-6, 192t-9, and TS IAV groups, and the numbers of IFN-γ secreting splenocytes were also increased in a dose-dependent manner. Finally, 192t-6, and 192t-9 can protect the mice against the challenge of homologous PR8 H1N1 virus and heterosubtypic H3N2 influenza virus. MiRNA targeted viruses 192t-6 and 192t-9 were significantly attenuated and showed the same virulence as TS IAV and played a role in the cross-protection.

## 1. Introduction

Influenza vaccine is the recommended approach to protect against influenza virus infection. Currently, influenza vaccines approved by the US FDA include inactivated influenza vaccine (IIV), recombinant influenza protein (RIP), and live attenuated influenza vaccine (LAIV) [1]. Studies have shown that inactivated influenza virus vaccine is wildly used and shows good safety and immunogenicity after immunization in humans [2,3,4]. LAIV, to a certain extent, has several advantages over IIV. Currently, live attenuated influenza vaccine has been approved for use in humans in USA, Russia, Europe, Canada, and Asia (Japan, South Korea, etc.) [5,6]. Several studies found that LAIVs have good immunogenicity, and can induce humoral immunity, especially mucosal immunity and cellular immunity to provide cross-protection against influenza virus infection and might provide better protection than IIV due to influenza virus antigenic drift and shift. It may be one of the reasons that LAIV could induce more effective secretory IgA responses [7]. The standard reverse genetics method can be used to generate live attenuated influenza vaccines. For example, LAIV can be constructed by truncating influenza virus genes or optimizing codon pairing [8,9,10]. Recently, there have been reports of LAIV based on miRNA mediated design [11,12,13,14].

MiRNA is a small non-coding RNA with a length of 20–22 nt, which is normally found in humans or animals, and can participate in the degradation and translational repression of target mRNA, and take part in viral infection or replication by targeting viral RNA [15]. High expression miRNA in human and mouse epithelial cells was screened by high throughput method, the hsa-miR-1-3p can be transfected into human lung cancer A549 cells and then infected with PR8 or H3N2 virus. Hsa-miR-1-3p can down-regulate NP gene expression and inhibit PR8 and H3N2 influenza viruses replication [15]. Some studies have shown that, if the expression of miRNA-485 in the host during a viral infection was inhibited, the replication ability of H5N1 virus in the host was reduced [16]. Moreover, through the method of high throughput sequencing, the miRNA which can reduce H7N9 reproduction rate in the host was screened. After the miRNA was transfected into A549 cells and infected by H7N9 virus, we found that inhibiting the hsa-miR-664-3p can obviously reduce H7N9 virus replication, or reduce H1N1 and H3N2 viruses replication [17], which indicated that miRNA plays an important regulatory role when the influenza virus attacks host cells. However, these miRNAs were only used for regulating virus vRNA to study the virus attenuated mechanism in these researches. These miRNAs were not as candidate target sites for LAIV.

Furthermore, it has been reported that other miRNA target site could be inserted into the genome of influenza virus to rescue the viral strains and achieve the goal of attenuating its pathogenicity. The miR-let-7b target site was inserted into the PB1 gene of H1N1 influenza virus, and the rescued virus strain was obviously attenuated in cells and animals [11]. In addition, miRNA target sites, such as miR-21 and miR-142 could also be inserted into the NP genome segment of influenza virus to regulate and weaken the replication ability of the influenza virus [12,14]. The miRNA-192-5p exists simultaneously in human and mouse epithelial cells [15], and is over-expressed in tumor cells such as gastric cancer cells and lung cancer cells [18]. Over-expression of miRNA-192 can also inhibit the replication of the HCV virus [19]. The miRNA-192-5p is both absent in MDCK cells and 293T cells in our preliminary experiments. Moreover, other study showed that when four perfect miRNA-192 target sites inserted into the HA genome of H5N1 virus, the recombinant virus was attenuated somehow [13]. Therefore, miRNA-192-5p could be used as a candidate for miRNA-based LAIV.

Our study mainly explored the different perfect miRNA-192-5p target sites which were inserted into the NP genome segment of the PR8 influenza virus, so as to study the virulence of miRNA targeted viruses, their pathogenicity on cells and animals, their immunogenicity in a mouse model, as well as the protective immunity during the homologous PR8 and heterosubtypic H3N2 viruses challenge.

## 2. Materials and Methods

### 2.1. Cells, Viruses, and Mice

MDCK cells (Madin Darby Canine Kidney) and 293T cells (Human embryonic Kidney) were purchased from ATCC, cultured with 10% FBS (Biowest S1810-500) in DMEM (Gibco) at 37 °C and 5% CO_2_ incubator. The mouse-adapted strains including A/Puerto Rico/8/34 (H1N1) (PR8) and A/Jingke/30/95 (H3N2) preserved by Shanghai Institute of Biological Products.

Specific pathogen free (SPF) BALB/c female mice at 6–8 weeks were purchased from Shanghai sepulch-pichai experimental animal center. All experiments involving animals were approved by the Animal Care Committee of the Shanghai Institute of Biological Products (Protocol Number: 17-1251). Each mouse was anesthetized intraperitoneally with 1% pentobarbital sodium at 60 mg/kg, and infected intranasally (i.n) with 20 μL virus at different concentrations. Weight changes and mortality were observed after immunization, and mice were sacrificed when they lost 25% of their initial starting weight. Lungs were harvested at 3 days after infection or challenge, and the virus titers were measured by 50% tissue culture infection dose (TCID_50_) and calculated by the Reed-Muench method. The inactivated virus was obtained by UV exposure at room temperature for 1 h. 

### 2.2. Plasmid Construction, Virus Rescue and Multicycle Growth Curve Analysis

A/Puerto Rico/8/1934 H1N1(PR8) viral segments cloned into pHW2000 vector have been described in our previous study [20]. The different perfect miRNA-192-5p(5′-GGCTGTCAATTCATAGGTCAG-3′) target sequences or scrambled control (5′-ATCGATAGGAATTTACCAAAT-3′) target sequences were inserted into the NP genome segment between the stop codon and duplicated packaging sequence [13]. Viruses were rescued by standard reverse genetics method, confirmed by sequencing, and quantified by plaque assay [12,14]. After viruses passing through chicken embryos for 10 generations, the viral RNAs were extracted and amplified by PCR assay and confirmed by sequencing.

MDCK and MDCK 192 cells transfected with miRNA-192 mimic in culture were infected with viruses at a MOI of 5, and DMEM medium containing 5 μg/mL TPCK trypsin (Sigma, T1246) was added. Virus supernatant was collected at 24 h, 48 h and 72 h after infection and stored at −80 °C.

### 2.3. MDCK Cells Transfection

MDCK cells with density of 60–70% were transfected with 50 nM miRNA mimic (GenePharma, Shanghai, China) or 50 nM miRNA negative control (GenePharma, Shanghai, China), and a mixture with 5 μL Lipofectamine 2000 reagent (Invitrogen, 11668-019). After transfection for 4 h, DMEM media containing 10% FBS was replaced. The RT-qPCR assay was performed to confirm the transfection efficiency. The second day, the transfected MDCK cells (MDCK 192 cells) were infected by viruses.

### 2.4. RT-qPCR

MDCK cells transfected with miRNA mimic and miRNA negative control for 24 h were extracted with Trizol Reagent (Invitrogen, USA) for total RNA. Reverse transcription was performed according to the Kit instructions (all-in-one ™ miRNA first-strand cDNA Synthesis Kit, GeneCopoeia). Quantitative PCR was performed using SYBR Green method (all-in-one ™ miRNA qPCR Kit, GeneCopoeia). Primer of miRNA-192-5p: 5′-cggCTGACCTATGAATTGACAGCC-3′ and Internal reference U6 primer: 5′-ATGGCCCCTGCGCAAGGA-3′. The comparative threshold cycle (Ct) method was used to quantify relative expression.

### 2.5. Immunization and Viral Challenge

For a homologous protection study, mice were i.n. immunized once with a total volume of 20 μL, with different doses of 192t-6, 192t-9 and TS IAV which was the positive control group (10 pfu, 100 pfu and 1000 pfu). The naïve (an unimmunized group), 100 pfu UV 192t-6 and 100 pfu UV 192t-9 were the control groups. Four weeks after immunization, mice were anesthetized and challenged i.n with 20 μL 10× LD_50_ A/Puerto Rico/8/1934 H1N1(PR8) virus. 

For a heterosubtypic protection study, mice were i.n immunized with 100 pfu 192t-6 and 100 pfu 192t-9, and naïve which was the control group. Four weeks after the immunization, mice were anesthetized and challenged i.n with 20 μL 5× LD_50_ A/Jingke/30/95 (H3N2) virus.

### 2.6. Enzyme-Linked Immunosorbent Assay (ELISA)

Four weeks after immunization, five mice were randomly selected from each group and sacrificed. Mouse lungs were collected homogenized in 1 mL of PBS by an electric homogenizer Tissuelyser-24 (Jingxin, Shanghai, China). The blood was placed overnight at 4 °C, spun at 6000× *g* for 10 min at 4 °C. Serum was collected, and antibodies in serum and lung homogenates were measured by ELISA [21]. The 5 μg/mL of inactivated whole-virion H1N1 vaccine was used as an antigen. The secondary antibodies were using the following antibodies: HRP-labeled sheep anti-mouse IgG, IgA, IgG1, IgG2a, and IgG2b (Southernbiotech). Ab-positive cut-off values were set as means + 2× SD of the naive group.

### 2.7. Enzyme-Linked Immune Spot Test (ELISpot)

Four weeks after immunization, five mice of each group were sacrificed to separate spleens by sterile forceps to prepare PBMC. The secretion of specific IFN-γ from mouse splenocytes was measured by ELISpot assay in previous study [22]. The 10 μg/mL of inactivated whole-virion H1N1 vaccine was used as a stimulant to detect specific IFN-γ secreting splenocytes and the number of splenocytes were 3 × 10^5^ per well.

### 2.8. Statistics

GraphPad Prism 6 software was used to perform statistical analyses. *T*-test was used for comparison between the two groups, and one-way ANOVA was used for comparison between multiple groups. *p* < 0.05 was statistically significant.

## 3. Results

### 3.1. Construction of Vaccines by Inserted with Different Perfect miRNA Target Sites

**Figure 1:**
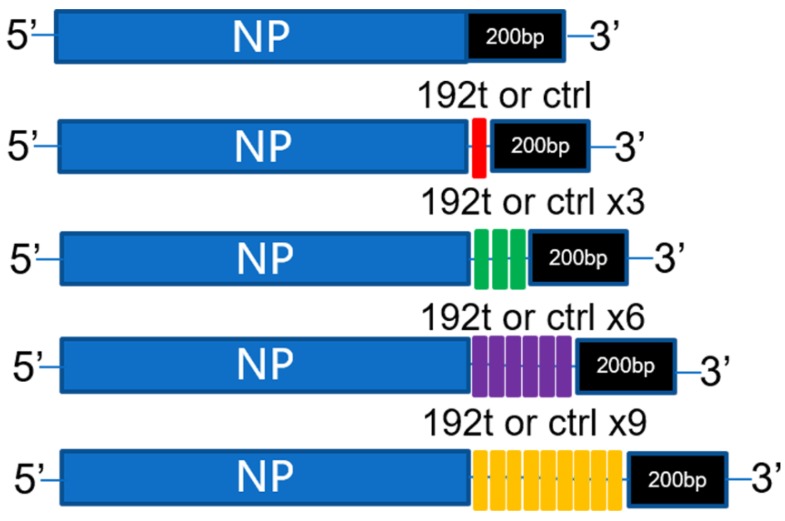
Generation of virus by insertion of different perfect miRNA target sites. Schematic illustrating NP and engineered NP viral segments. The downstream of the NP genome stop codon but upstream of the duplicated packaging sequence were inserted by different perfect miRNA-192-5p target sites or control target sites.

In this study, different perfect miRNA-192-5p target sites were attempted to be inserted into the NP genome of influenza virus (Figure 1). Downstream of the NP genome stop codon but upstream of the duplicated packaging sequence were inserted by different perfect miRNA-192-5p target sites or control target sites, the seven other unmodified genes were constructed as described in Materials and Methods. The viruses were rescued by standard reverse genetics method in 293T cells, while 293T cells could not express miR-192 confirmed by RT-qPCR assay. The viruses rescued were confirmed by sequencing. After viruses passing through chicken embryos for 10 generations, the sequencing results indicated that no mutation in the NP genome segment of the viruses was found, which also confirmed the stability of the viruses.

### 3.2. Viruses with Different Perfect miRNA Target Sites Inserted into NP Genome Segment Demonstrate Variable Attenuations in Cells

**Figure 2:**
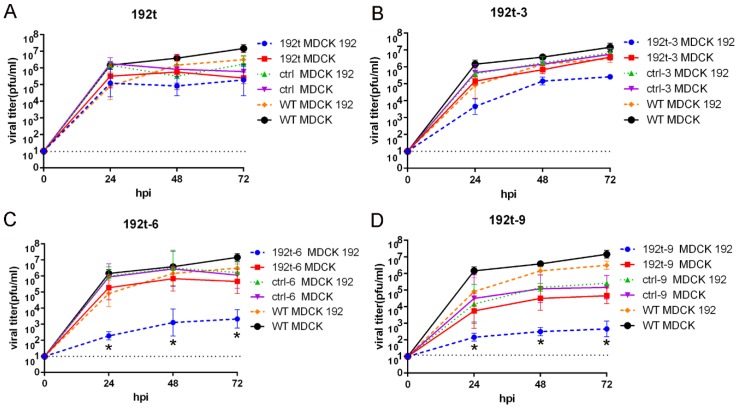
miR-192 targeted viruses of 192t-6 and 192t-9 most effectively reduce viral replications in cells. MDCK and MDCK192 cells were infected with miR-192 targeted viruses and all control viruses at a MOI of 5, (**A**) WT, 192t and ctrl, (**B**) WT, 192t-3 and ctrl-3, (**C**) WT, 192t-6 and ctrl-6, (**D**) WT, 192t-9 and ctrl-9. Virus supernatant was collected at 24 h, 48 h, and 72 h after infection, and quantified by plaque assay. Data points at the indicated times were performed in triplicate; error bars represent standard deviation. * *p* < 0.05, compared to WT MDCK 192 group. Data are representative of two independent experiments. Dotted line represents limit of detection. Abbreviation: WT, wild type virus; hpi, hours post-infection.

To determine the replication of the viruses with different perfect miRNA target sites inserted into NP genome segment, we carried out analysis of the multicycle growth curve. However, the expression of miRNA-192-5p in MDCK cells was absent, then MDCK cells were engineered by transfection of 50 nM miRNA-192-5p mimic to overexpress miRNA-192-5p (MDCK 192 cells), and the RT-qPCR assay was performed to confirm the expression of miRNA-192-5p in MDCK cells after the transfection. We observed that the 192t virus with one perfect miRNA target site showed similar levels of replication in both MDCK and MDCK 192 cells (Figure 2A). 192t-3 virus with three perfect miRNA target sites were attenuated in MDCK 192 cells, with a one to two log reduction in viral titers as compared to in MDCK cells (Figure 2B). Significant attenuated effects were observed in 192t-6 virus with six perfect miRNA target sites and 192t-9 virus with nine perfect miRNA target sites, with a two to three log reduction in viral titers (Figure 2C,D). As expected, WT virus and all control viruses showed similar replication kinetics in MDCK and MDCK 192 cells. 

### 3.3. Viruses with Different Perfect miRNA Target Sites Inserted into NP Genome Segment Demonstrate Variable Attenuations in Mice

**Figure 3:**
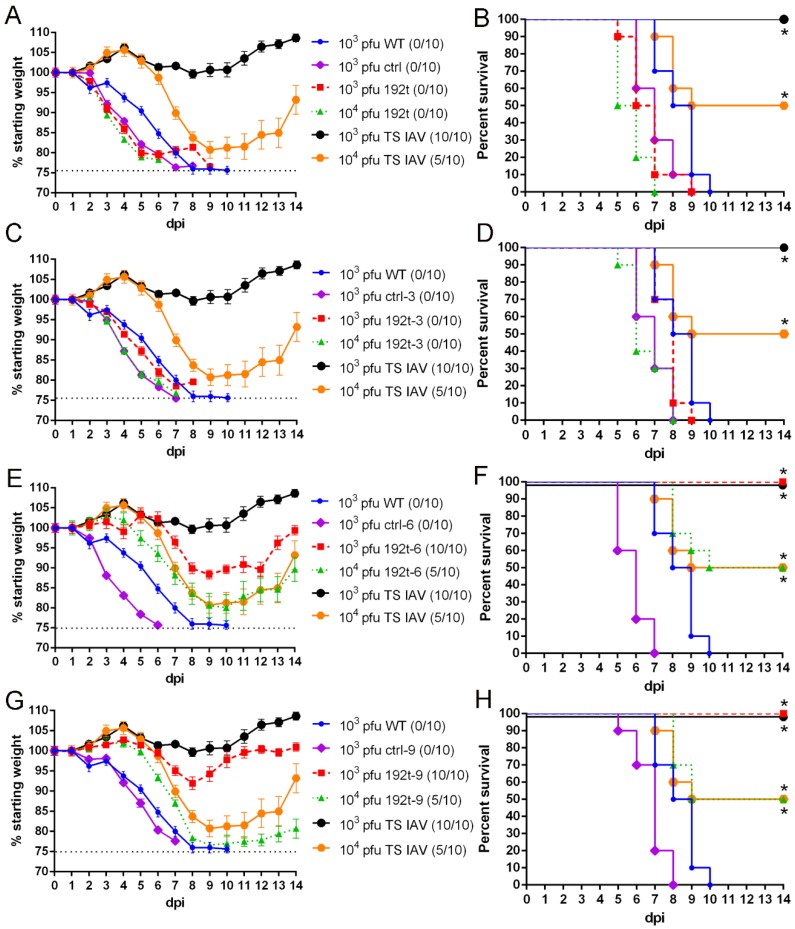
miR-192 targeted viruses of 192t-6 and 192t-9 are attenuated in mice. Mice were i.n. infected with 10^3^ plaque-forming units (pfu) or 10^4^ pfu of miR-192 targeted viruses, and with 10^3^ pfu of ctrl, ctrl-3, ctrl-6, ctrl-9 and WT. Bodyweight changes (**A**,**C**,**E**,**G**) and survival rates (**B**,**D**,**F**,**H**) of mice were measured daily for two weeks after infection. * *p* < 0.05, compared to the WT group. Abbreviation: dpi, days post-infection.

We next determined the virulence of miR-192 targeted viruses *in vivo*. Meanwhile, a temperature-sensitive (TS IAV) constructed from PR8 virus was used as a positive control [23]. Mice were i.n. infected with 10^3^ plaque-forming units (pfu) or 10^4^ pfu of miR-192 targeted viruses, and with 10^3^ pfu of all control viruses and WT. Mice infected with 10^3^ pfu 192t and 10^4^ pfu 192t demonstrated weight loss and succumbed to the infection after six days from the infection. The same result was also observed in mice infected with 10^3^ pfu 192t-3 and 10^4^ pfu 192t-3 (Figure 3A–D). Importantly, all mice survived after infection with 10^3^ pfu 192t-6 and 10^3^ pfu TS IAV, while all mice infected with 10^4^ pfu 192t-6 and 10^4^ pfu TS IAV were demonstrating 50% survival and weight loss (Figure 3E,F). Mice infected with 10^3^ pfu 192t-9 also showed the same result as that with 10^3^ pfu 192t-6, and mice infected with 10^4^ pfu 192t-9 showed 50% survival and weight loss, the same result was found in mice with 10^4^ pfu TS IAV infection (Figure 3G,H). In contrast, all mice with infection of WT and all control viruses showed weight loss and succumbed to the infection within ten days after the infection. The results of the viral growth 3 days after infection of the viruses were consistent with the weight loss and survival data, and analysis of the lung viral titers also demonstrated a selective block of the viruses replications in 10^3^ pfu 192t-6, 10^3^ pfu 192t-9 and 10^3^ pfu TS IAV groups, importantly, even the viral titers of 10^4^ pfu 192t-6, 10^4^ pfu 192t-9 and 10^4^ pfu TS IAV were lower than that of all control viruses and WT groups. It is possible that the mice infected with 192t-6, 192t-9 and TS IAV showed relatively slow infection symptoms, and partial infection only occurred 6 to 7 days after the infections. Therefore, no virus infection symptoms were observed, and we detected a significant reduction in the lung virus titers 3 days after the infections. As expected, mice infected with 10^3^ pfu 192t and 10^4^ pfu 192t, also with 10^3^ pfu 192t-3 and 10^4^ pfu 192t-3 showed the same high viral titers as the groups with WT and all control viruses (Table 1).

Taken together, these results suggested that the virulence of 192t-6 and 192t-9 were attenuated in cells and animals.

### 3.4. MiRNA Targeted Viruses of 192t-6 and 192t-9 Provide Protection from Homologous Virus Infection

**Figure 4:**
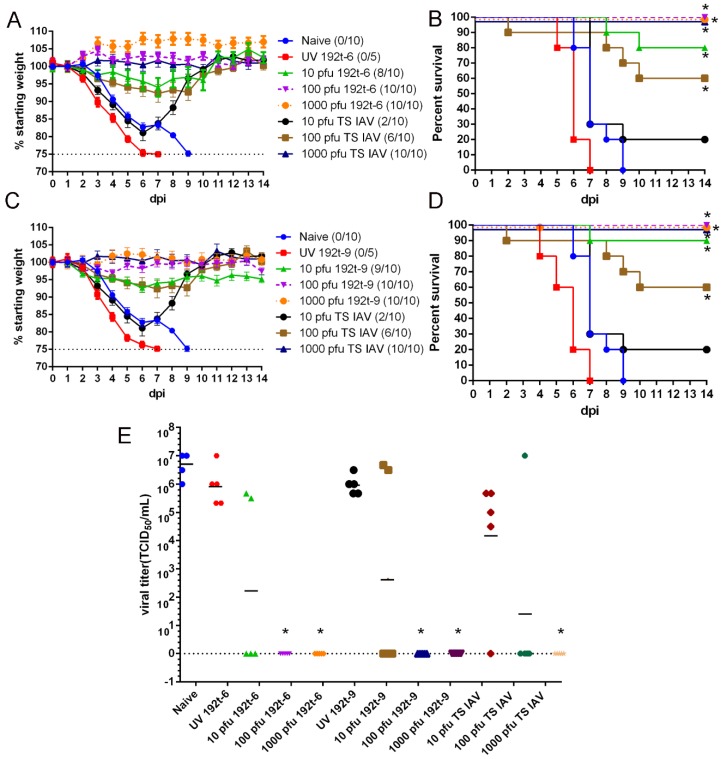
miRNA targeted viruses of 192t-6 and 192t-9 provide protection against homologous virus challenge. Mice were immunized i.n. once with various doses of 192t-6, 192t-9 and TS IAV which was as the positive control group, and naive, UV 192t-6 and UV192t-9 which were as control groups. Four weeks after immunization, mice were challenged with a high lethal dose of 10× LD_50_ PR8 influenza virus. Bodyweight changes (**A**,**C**) and survival rates (**B**,**D**) of mice were measured daily for two weeks after challenge. * *p* < 0.05, compared to the naive group. (**E**) The lung viral titers of all mice were determined by TCID_50_ on three days after the challenge. Data shown as mean, *n* = 5 per group. Each sample was tested in triplicates. Dotted line represents limit of detection. * *p* < 0.05, compared to the naive group.

To measure whether vaccination with 192t-6 and 192t-9 could protect mice from lethal dose of homologous virus challenge, mice were all immunized i.n. once with various doses of 192t-6, 192t-9 and TS IAV which was the positive control group, as well as naive, UV 192t-6 and UV 192t-9 which were the control groups. Four weeks after immunization, all mice were challenged with a lethal dose of PR8 virus. As expectedly, the mice in the naïve, UV 192t-6 and UV 192t-9 groups failed to provide any protection. We observed that the high dosage group of TS IAV could provide full protection, while the groups of the low and medium dosage of TS IAV only provided 20% and 60% protection. However, all mice immunized with the medium and high dosage of 192t-6 and 192t-9 could provide full protection. Meanwhile, the low dosage of 192t-6 and 192t-9 provided 80% and 90% protection, respectively (Figure 4A–D).

Three days after the challenge, the lung virus titers in the groups of the low and medium dosage of TS IAV, and in the groups of the low medium dosage of 192t-6 and 192t-9 could be detected, but all lower than that of the naïve, UV 192t-6 and UV 192t-9 groups. However, the results of the lung viral titers demonstrated a selective block of the virus replication in the group of the high dosage of TS IAV immunization. The lung viral titers of the groups in immunization with the medium and high dosage of 192t-6 and 192t-9 showed the same result as that with the high dosage of TS IAV (Figure 4E). These data indicated that vaccination with 192t-6, 192t-9 and TS IAV induced protection during challenge with homologous virus and reduced the viral load in the lungs after the challenge.

### 3.5. MiRNA Targeted Viruses of 192t-6 and 192t-9 Provide Protective Immunity during Lethal Challenge of Homologous Virus

Having verified the provided protection from the vaccination with 192t-6,192t-9 and TS IAV, we next assessed the protective immunity response after the vaccinations. Mice were immunized with various doses of 192t-6, 192t-9 and TS IAV, and also immunized with the naïve, UV 192t-6 and UV 192t-9. Four weeks after immunization, 5 mice in each group were sacrificed, and the titers of virus-specific IgG and IgG isotypes in serum, virus-specific IgA in lung homogenate were assessed. The IFN-γ secretion of splenocytes of immunized mice was detected by ELISpot assay. 

The results demonstrated that all groups immunized with 192t-6, 192t-9 and TS IAV could induce significant serum antibody response and high levels of mucosal IgA in a dose-dependent manner, with the high immune dose group in high antibody titer. The titers of specific antibodies were positively correlated with the above protective effect. To further determine the Th bias of the IgG specific humoral immune response, we observed the IgG isotypes in serum. IgG1, IgG2a and IgG2b antibody titers were enhanced in the groups of 192t-6, 192t-9 and TS IAV, meanwhile IgG2a titers were obviously higher than IgG1 titers. Thus, vaccination with 192t-6, 192t-9 and TS IAV mainly produced a Th1 type immune response. Conversely, the naïve, UV 192t-6 and UV 192t-9 groups failed to induce specific antibody responses (Table 2).

**Figure 5:**
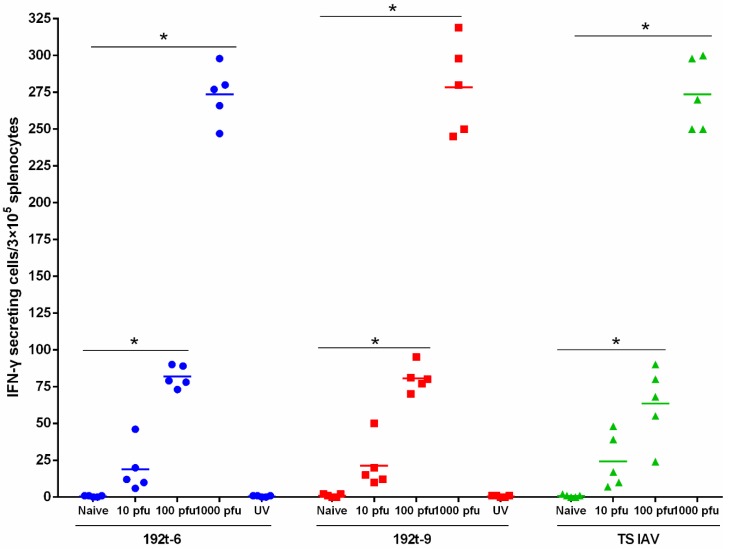
miRNA targeted viruses of 192t-6 and 192t-9 enhance cellular immune response. Mice were immunized i.n. once with various doses of 192t-6, 192t-9 and TS IAV which was as the positive control group, and naive, UV 192t-6 and UV 192t-9 which were as control groups (*n* = 5 per group). The number of IFN-γ secreting splenocytes of immunized mice was measured by ELISpot assay four weeks after immunization. Data shown as mean numbers of spot-forming cells (SFCs). Each sample was tested in triplicates. * *p* < 0.05, compared to the naive group.

The cellular immune response was assessed by an IFN-γ ELISpot assay, we found the vaccination groups of 192t-6, 192t-9 and TS IAV increased a number of IFN-γ secreting splenocytes in a dose-dependent manner, compared with the naïve, UV 192t-6 and UV 192t-9 groups (*p* < 0.05). Taken together, vaccination with 192t-6, 192t-9 and TS IAV enhanced systemic and mucosal antibody, as well as cellular immune responses (Figure 5).

### 3.6. MiRNA Targeted Viruses of 192t-6 and 192t-9 Provide Protection Against Heterosubtypic Virus Challenge

**Figure 6:**
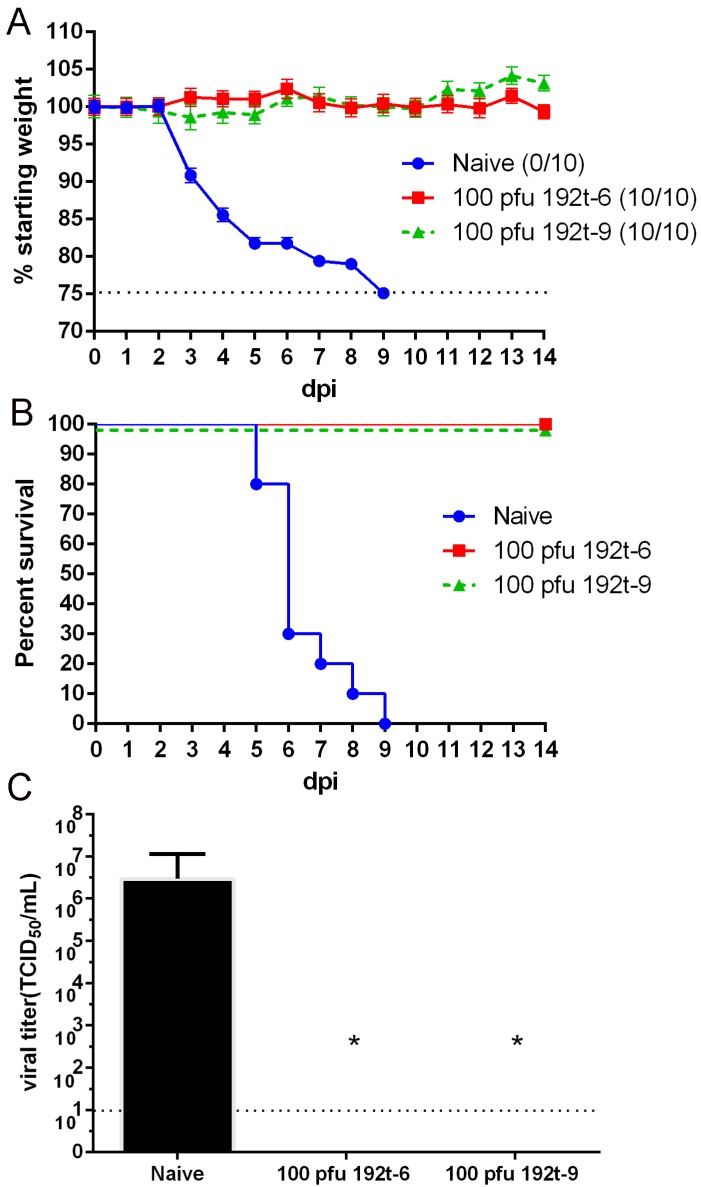
miRNA targeted viruses of 192t-6 and 192t-9 provide protection against heterosubtypic virus challenge. Mice were immunized i.n. once with 192t-6, 192t-9 and naïve, and challenged with a lethal dose of 5× LD_50_ H3N2 influenza virus four weeks after immunization. Bodyweight changes (**A**) and survival rates (**B**) of mice were measured daily for two weeks after challenge. (**C**) Mice in each group were detected at three days after the challenge by TCID_50_ assay. Data shown as mean ± SD. *n* = 5 per group. Each sample was tested in triplicates. Dotted line represents limit of detection. * *p* < 0.05, compared to the naive group.

In order to determine if 192t-6 and 192t-9 could provide cross-protection, mice were immunized with 192t-6 and 192t-9 respectively and challenged with 5× LD_50_ of the H3N2 avian influenza virus at 4 weeks after immunization. As shown in Figure 6, all mice vaccinated with 192t-6 and 192t-9 were protected from heterologous virus challenge demonstrating 100% survival and no significant weight loss, in contrast with the fact that the naïve group succumbed to the infection. Three days after the challenge, the lung virus titers in the 192t-6 and 192t-9 groups were significantly lower than that in the unvaccinated group (*p* < 0.05), which were consistent with the protective effect. In conclusion, miRNA targeted viruses of 192t-6 and 192t-9 not only provided protection from homologous virus infection but also provided protection against heterosubtypic virus challenge. 

## 4. Discussion

MiRNA is a small non-coding single-stranded RNAs that can exist in healthy human or animal and participate in gene regulation after post-transcription. Some studies have shown that miRNA also exists in cancers, of which the miRNA-192 can be highly expressed in ovarian carcinomas of the mucinous subtype [24]. The miRNA-192 exists in different tumor cells, including cancer of the stomach, hepatocellular carcinoma, etc., and can promote the proliferation and apoptosis function of B-cell precursor cell line (NALM-6) [25]. MiRNA-192 also can be used as a new biomarker of the acute liver injury induced by acetaminophen, and over-expression of the miRNA-192 can inhibit the replication of HCV virus [19]. Moreover, it was also found that the decrease of expression level of miR-192 was related to the increase of HBV DNA level in serum of HBV patients. MiR-192-5p presents in HBsAg particles, and the higher level of the HBeAg is, the higher expression level of miR-192-5p [26,27]. The miR-192 is also correlated with the porcine circovirus-associated diseases [28].

However, miRNA-192-5p exists simultaneously in human and mouse lung epithelial cells [15], and moreover, it is both absent in MDCK cells and 293T cells. Relevant study has demonstrated that miRNA-192-5p is over-expressed in A549 cells, which are human embryonic lung tumor cells and can be used as a cell model for influenza virus infection in humans, and it might be the miRNA which could potentially restrict influenza virus replication in humans and mice, and be used as a candidate for miRNA-based LAIV across susceptible hosts [13,29]. In recent years, some researchers have used the miRNA-based strategy to design live attenuated influenza vaccines [13,14]. When the virus whose genome has been inserted with the respective miRNA target sites infects the cells or mice, the specific viral NP mRNA will be recognized and targeted by the cellular miRNA, and then cleaved by Argonaute 2 protein. The viral gene will also be post-transcriptionally repressed by micro-ribonucleoproteins, thus the miRNA-based virus could be attenuated somehow [11,30].

Influenza virus NP gene as a major internal virion protein, encapsulates the viral genome, plays an important role in the influenza virus life cycle [31], and participates in the whole process of the influenza virus replication. MiRNA can participate in the process of virus infection or replication through targeting viral RNA. Thus, in our study, different perfect miRNA-192 target sites (segment 1, 3, 6, and 9) were inserted into the PR8 NP genome. In addition, the expression of miRNA-192-5p gene in MDCK cells was absent, so MDCK cells were engineered by transfection to overexpress miRNA-192-5p (MDCK 192 cells) which were infected by miR targeted viruses. The results of virulence and pathogenicity in cells and mice showed that the viruses inserted with different perfect miRNA target sites, were related with the attenuated effect, as the viruses inserted with the 6 and 9 perfect miR target sites have more attenuated effect than the viruses inserted with the 1 and 3 perfect miR target sites, but the virus inserted with 9 perfect miR target sites has similar attenuated effect in cells and animals experiments with the virus inserted with 6 perfect miR target sites. Along with the increase in virus inserted miR repeated target sites, the attenuated effect is strengthening, but when increased to 6 perfect miR target sites, the attenuated effect reaches a plateau, and will not be enhanced with the increase of miR repeated target sites. Similar results have also been shown in the West Nile virus (WNV), the strains inserted with 3 perfect miR target sites was slightly better than insertion of 2 perfect miR target sites, and insertion of 2 different miRNA target sites was stronger than insertion of 1 miR target site [32]. 

After intranasal immunization, LAIV could induce strong humoral immunity, mucosal immunity and cellular immunity [7]. LAIV could induce high levels of influenza virus-specific antibody which was closely related to protection against influenza virus infection [33]. The results of this study showed that 192t-6, 192t-9 and temperature-sensitive TS IAV could promote the production of specific antibodies, not only increasing the IgG humoral antibody level, but also increasing the IgA mucosal antibody level, and the immunized mice could resist the challenge of homologous PR8 virus, which indicated that IgG and IgA antibodies played an important role in the immune protection provided by 192t-6 and 192t-9 viruses. In addition, it has also been proved that not only IgG antibody and IgA antibody play a role in the immune protection provided by LAIV, but also the induced IgG antibody subtypes, including IgG1 and IgG2a antibodies can mediate the immune response and are involved in the protective efficacy. Of which IgG2a antibody is involved in Th1 type immune response, which can accelerate the clearance of influenza virus and resist influenza virus infection. IgG1 antibody regulates Th2 type immune response and neutralizes virus particle [20]. Studies have shown that TS IAV mainly produces IgG2a antibody which participates in Th1 type immune response, and can stimulate lymphocyte secreted IFN-γ [20], which plays an important role in B cell and T cell development, bridge innate and adaptive immunity [34]. In this study, it was also found that, the levels of serum IgG2a antibody were higher than that of serum IgG1 and IgG2b antibodies after immunizing 192t-6, 192t-9 and TS IAV. The results indicated that 192t-6 and 192t-9 mainly produced Th1 type immune response which was stronger than Th2 type immune response. At the same time, after immunization of 192t-6 and 192t-9, the mice lymphocytes were stimulated to secrete IFN-γ, with good immunogenicity. In general, 192t-6 and 192t-9 could induce the mice to produce the humoral, mucosal and cellular immune responses.

A large number of studies have shown that LAIV can induce a cross-protective response against both homogenous and heterologous viruses challenge [7,35]. TS IAV is safe and effective, and can induce humoral, mucosal and cellular immunity which can protect humans from influenza virus infection [23,36,37]. The results in our study also found that 192t-6, 192t-9 and TS IAV could protect mice from PR8 homologous virus infection and reduce the replication of influenza virus in mice, and 192t-6 and 192t-9 had similar protective effects on mice as TS IAV. At the same time, the mice immunized with 192t-6 and 192t-9 also can completely combat the challenge of heterologous H3N2 virus, while eliminating the virus titers in mice. However, the research was aimed to study the attenuation of miRNA targeted viruses, while other study has shown that TS IAV vaccine could protect mice against homologous and heterologous viruses infection [38], therefore, TS IAV was not included in the heterologous challenge studies in the study.

Nevertheless, relevant studies have demonstrated that NP-21t strain that was rescued by the NP genome segment inserted with miR-21 target sites could attenuate influenza pathogenicity significantly, and also could induce specific antibody response after immunization in mice with the same as FDA approved CA IAV, which can completely protect mice from a PR8 virus challenge [14]. Similarly, the virus rescued by NP genome inserted with miRNA-93 target site could also protect mice from a lethal homologous influenza virus challenge [39]. A study has also shown that an H5N1 virus strain rescued with its HA genome inserted with miRNA-192 target sites was attenuated in cells or mice [13]. 

In this study, we demonstrated that the miRNA target sites were inserted into PR8 NP genome segment to rescue the attenuated viruses, as the virus 192t-6 inserted with 6 perfect miRNA target sites and the virus 192t-9 inserted with 9 perfect miRNA target sites could attenuate pathogenicity significantly, which was similar to the pathogenicity of temperature-sensitive TS IAV. Moreover, it was proved that miRNA-based viruses of 192t-6 and 192t-9 could protect mice from challenge of homologous and heterosubtypic influenza viruses and provide cross-protective effect. It suggested that miRNA-based strategy could be used in the research and development of a live attenuated influenza vaccine.

## Figures and Tables

**Table 1:** The Lung virus titer and the mortality of mice by infection of miRNA targeted viruses.

Group	Dose (pfu/20 μL)	Lung Virus Titer ^a^ (Log_10_TCID_50_/mL)	Survival Rate (No. of Survivors/No. Tested)
192t	10^3^	5.67 ± 0.89	0/10
	10^4^	6.60 ± 0.28	0/10
192t-3	10^3^	5.13 ± 0.46	0/10
	10^4^	6.33 ± 0.34	0/10
192t-6	10^3^	ND	10/10
	10^4^	2.42 ± 0.49	5/10
192t-9	10^3^	ND	10/10
	10^4^	2.44 ± 0.16	5/10
TS IAV	10^3^	ND	10/10
	10^4^	2.39 ± 0.08	5/10
ctrl	10^3^	6.10 ± 0.42	0/10
ctrl-3	10^3^	5.80 ± 0.76	0/10
ctrl-6	10^3^	5.83 ± 0.24	0/10
ctrl-9	10^3^	5.47 ± 0.49	0/10
WT	10^3^	5.90 ± 0.36	0/10

Mice were i.n. infected with 10^3^ plaque-forming units (pfu) or 10^4^ pfu of miR-192 targeted viruses, and with 10^3^ pfu of ctrl, ctrl-3, ctrl-6, ctrl-9, and WT. The lung viral titers from 5 mice in each group were detected three days post-infection by TCID_50_ assay. The survival rates of mice two weeks post-infection were determined. Abbreviation: ND, not detected. ^a^ Results are expressed as mean ± SD of five tested mice in each group.

**Table 2:** Serum and mucosal antibody responses in mice by intranasal administration of 192t-6 or 192t-9 or TS IAV vaccine.

Group	Virus-*S*pecific Antibody Titer (ELISA, 2*^n^*) ^a^				
	Serum IgG	Lung Homogenate IgA	Serum IgG1	Serum IgG2a	Serum IgG2b
10 pfu 192t-6	16.8 ± 2.17	7.6 ± 0.89	12.8 ± 1.79	14.4 ± 0.89	13.0 ± 1.23
100 pfu 192t-6	17.6 ± 0.89	10.2 ± 0.84	16.0 ± 0.71	17.6 ± 0.89	16.4 ± 0.89
1000 pfu 192t-6	20.8 ± 0.45	13.8 ± 0.96	17.2 ± 1.10	18.2 ± 0.45	17.2 ± 2.17
10 pfu 192t-9	15.4 ± 4.10	7.2 ± 0.84	12.0 ± 3.00	13.4 ± 1.14	12.0 ± 2.12
100 pfu 192t-9	17.4 ± 1.14	10.4 ± 0.55	16.8 ± 1.10	17.2± 1.10	15.4 ± 1.14
1000 pfu 192t-9	20.4 ± 0.55	13.0 ± 1.58	17.4± 0.55	19.0 ± 0.71	16.8 ± 1.79
10 pfu TS IAV	9.2 ± 3.27	5.2 ± 0.84	8.8 ± 4.44	11.8 ± 1.79	9.2 ± 4.15
100 pfu TS IAV	11.6 ± 3.91	8.6 ± 1.52	10.8 ± 2.95	13.2 ± 1.10	11.2 ± 1.30
1000 pfu TS IAV	19.3 ± 0.58	12.2 ± 1.30	14.2 ± 1.10	14.2 ± 1.10	13.4 ± 1.14
UV 192t-6	2.4 ± 0.55	3.0 ± 0.71	2.8 ± 0.84	2.6 ± 0.89	2.8 ± 0.84
UV 192t-9	2.4 ± 0.55	2.8 ± 0.45	3.0 ± 0.71	3.2 ± 0.84	3.4 ± 0.89
Naive	— ^b^	—	—	—	—

Mice were i.n. immunized once with various doses of 192t-6, 192t-9 and TS IAV vaccine. The naïve (an unimmunized group), 100 pfu UV 192t-6 and 100 pfu UV 192t-9 were as the control groups. Four weeks after the immunization, serum and lung homogenate specimens of 5 mice in each group were prepared. The titers of virus-specific IgG and specific IgG1, IgG2a, IgG2b in serum and virus-specific IgA in lung homogenate were determined by ELISA. ^a^ Results from five tested mice in each group are expressed as mean ± SD. ^b^ ND, not detected.
